# Supplementary material for: Comparative Effectiveness of First-Line Selpercatinib versus Standard Therapies in Patients with RET-Activated Cancers: An Exploratory Interpatient Analysis of LIBRETTO-001
Source: Cancers (Basel). 2023 Dec 27;16(1):140. doi: 10.3390/cancers16010140 (PMC10778524; doi:10.3390/cancers16010140)
Supplement: Supplementary file 1 [file cancers-16-00140-s001.zip › cancers-2778599-supplementary.pdf]

Supplementary Materials

Table S1. Treatment regimens of pre-treated patients with NSCLC, MTC, and TC in 1L

**Standard therapy regimens in 1L NSCLC (N = 247, pre-matching)**	
**Regimen**	**n (%)**
Chemotherapy	181 (73.3)
Chemotherapy + Anti-PD1/PD-L1	40 (16.2)
Anti-PD1/PD-L1 only	14 (5.7)
MKI/TKI	10 (4.1)
Not known/missing	2 (0.8)

**Regimen**	**n (%)**
Vandetanib	92 (67.7)
Cabozantinib	44 (32.4)

**Regimen**	**n (%)**
Radioactive iodine (RAI)	21 (47.7)
MKI	13 (31.7)
MKI + RAI	2 (4.9)
Chemotherapy	2 (4.9)
Chemotherapy + other	2 (4.9)
Everolimus	1 (2.4)

Abbreviations: MKI, multikinase inhibitor; MTC, medullary thyroid cancer; N, total number of patients; NSCLC, non-small cell lung cancer; PD1/PD-L1, programmed death-1/programmed death ligand-1; TC, thyroid cancer; TKI, tyrosine kinase inhibitor.

Table S2. Baseline characteristics among patients with *RET* fusion-positive aNSCLC – Subgroup 1

**(n [%]) unless specified**	**Pre-matched**			**Genetic matching**		
	**Selpercatinib arm (n = 69)**	**Comparator arm (n = 71)**	**p-value**	**Selpercatinib arm (n = 68)**	**Comparator arm (n = 68)**	**p-value**
Age at index date, year						
Mean (SD)	62.1 (13.0)	59.2 (11.3)	.1690	62.2 (13.1)	61.9 (10.7)	.8995
Sex						
Female	43 (62.3)	40 (55.6)		43 (63.2)	41 (60.3)	
Male	26 (37.7)	32 (44.4)	.4146	25 (36.8)	27 (39.7)	.7242
Race						
White	48 (69.6)	32 (44.4)		47 (69.1)	49 (72.1)	
Black/African American	4 (5.8)	7 (9.7)		4 (5.9)	4 (5.9)	
Asian	13 (18.8)	30 (41.7)		13 (19.1)	13 (19.1)	
Other	4 (5.8)	3 (4.2)	**.0149**	4 (5.9)	2 (2.9)	.8712
Time from initial diagnosis to 1L start						
Q1	1 (1.5)	33 (45.8)		2 (2.9)	2 (2.9)	
Q2	6 (8.7)	21 (29.2)		14 (20.6)	14 (20.6)	
Q3	23 (33.3)	8 (11.1)		26 (38.2)	27 (39.7)	
Q4	39 (56.5)	9 (12.5)		26 (38.2)	25 (36.8)	
Unknown	0 (0.0)	1 (1.4)	**<.0001**	0 (0.0)	0 (0.0)	.9980
Stage at initial diagnosis						
Stage I-IIIA	3 (4.4)	2 (2.8)		3 (4.4)	3 (4.4)	
Stage IIIB-IV	65 (94.2)	70 (97.2)		65 (95.6)	65 (95.6)	
Unknown	1 (1.5)	0 (0.0)	.5163	0 (0.0)	0 (0.0)	1.0000
ECOG Performance Status						
0/1	65 (94.2)	71 (98.6)		64 (94.1)	64 (94.1)	
2	4 (5.8)	1 (1.4)	.1571	4 (5.9)	4 (5.9)	1.0000
Smoking Status, no	48 (69.6)	48 (66.7)	.7121	47 (69.1)	47 (69.1)	1.0000
Brain metastases, yes	16 (23.2)	23 (31.9)	.2453	16 (23.5)	14 (20.6)	.6792

Abbreviations: ECOG, European Cooperative Oncology Group; n, number of patients in sub-group; Q1, 1st quartile; Q2, 2nd quartile; Q3, 3rd quartile; Q4, 4th quartile; 1L, first-line therapy; SD, standard deviation.

Notes: Statistical comparisons between treatment arms were computed using t-tests for continuous variables and Chi-square and Fisher's exact test for categorical variables.

Table S3. Baseline characteristics among patients with *RET* fusion-positive aNSCLC – Subgroup 2

(n [%]) unless specified	Pre-matched			Genetic matching		
	**Selpercatinib arm (n = 69)**	**Comparator arm (n = 40)**	**p-value**	**Selpercatinib arm (n = 68)**	**Comparator arm (n = 68)**	**p-value**
Age at index date, year						
Mean (SD)	62.1 (13.0)	59.8 (9.8)	.3527	62.2 (13.1)	59.2 (12.2)	.1775
Sex						
Female	43 (62.3)	17 (42.5)		43 (63.2)	31 (45.6)	
Male	26 (37.7)	23 (57.5)	**.0450**	25 (36.8)	37 (54.4)	**.0388**
Race						
White	48 (69.6)	19 (47.5)		47 (69.1)	52 (76.5)	
Black/African American	4 (5.8)	4 (10.0)		4 (5.9)	4 (5.9)	
Asian	13 (18.8)	16 (40.0)		13 (19.1)	6 (8.8)	
Other	4 (5.8)	1 (2.5)	.0582	4 (5.9)	6 (8.8)	.3573
Time from initial diagnosis to 1L start						
Q1	1 (1.5)	10 (25.0)		1 (1.5)	9 (13.2)	
Q2	6 (8.7)	16 (40.0)		6 (8.8)	6 (8.8)	
Q3	23 (33.3)	9 (22.5)		23 (33.8)	25 (36.8)	
Q4	39 (56.5)	5 (12.5)	**<.0001**	38 (55.9)	28 (41.2)	**.0460**
Stage at initial diagnosis						
Stage I-IIIA	3 (4.4)	0 (0.0)		3 (4.4)	0 (0.0)	
Stage IIIB-IV	65 (94.2)	40 (100.0)		65 (95.6)	68 (100.0)	
Unknown	1 (1.5)	0 (0.0)	.3001	0 (0.0)	0 (0.0)	.0799
ECOG Performance Status						
0/1	65 (94.2)	38 (95.0)		64 (94.1)	64 (94.1)	
2	4 (5.8)	2 (5.0)	.8604	4 (5.9)	4 (5.9)	1.0000
Smoking Status, no	48 (69.6)	23 (57.5)	.2027	47 (69.1)	47 (69.1)	1.0000
Brain metastases, yes	16 (23.2)	13 (32.5)	.2890	16 (23.5)	13 (19.1)	.5300

Abbreviations: ECOG, European Cooperative Oncology Group; n, number of patients in sub-group; Q1, 1st quartile; Q2, 2nd quartile; Q3, 3rd quartile; Q4, 4th quartile; 1L, first-line therapy; SD, standard deviation.

Notes: Statistical comparisons between treatment arms were computed using t-tests for continuous variables and Chi-square and Fisher's exact test for categorical variables.

Table S4. Genetic matching investigator-assessed outcomes among patients with *RET* fusion-positive aNSCLC – Subgroups 1 and 2

	**Subgroup 1**			**Subgroup 2**		
	**Selpercatinib arm (n = 68)**	**Comparator arm (n = 68)**	**p-value**	**Selpercatinib arm (n = 68)**	**Comparator arm (n = 68)**	**p-value**
**TTD**						
Median (95% CI), month	25.3 (17.4, 32.6)	3.9 (2.9, 5.7)		25.3 (17.4, 32.6)	1.4 (1.4, 4.9)	
HR (95% CI)	0.20 (0.13, 0.30)		**<.0001**	0.14 (0.09, 0.22)		**<.0001**
**TTNT-D**						
Median (95% CI), month	31.0 (21.5, 41.7)	16.7 (13.8, 23.7)		31.0 (21.5, 41.7)	2.5 (2.5, 7.5)	
HR (95% CI)	0.65 (0.43, 0.97)		**.041**	0.14 (0.09, 0.22)		**<.0001**
**TTP**						
Median (95% CI), month	22.0 (13.8, 28.4)	16.7 (13.8, 23.7)		22.0 (13.8, 28.4)	2.5 (2.5, 4.7)	
HR (95% CI)	0.82 (0.55, 1.21)		0.365	0.17 (0.11, 0.26)		**<.0001**
**ORR**						
N (%)	58 (85.3)	16 (23.5)		58 (85.3)	13 (19.1)	
OR (95% CI)	18.9 (7.9, 45.2)		**<.0001**	24.5 (9.9, 60.6)		**<.0001**

Abbreviations: CI, confidence interval; HR, hazard ratio; n, number of patients in sub-group; TTD, time to treatment discontinuation; TTNT-D, time to next treatment or death; TTP, time to progression.

Table S5. Baseline characteristics among patients with *RET*-mutated MTC – Subgroup 1

(n [%]) unless specified	Pre-matched			Genetic matching		
	Selpercatinib arm (n = 116)	Comparator arm (n = 73)	p-value	Selpercatinib arm (n = 113)	Comparator arm (n = 113)	p-value
Age at index date, year			.8969			.9562
Mean (SD)	55.8 (15.5)	55.5 (15.0)		55.7 (15.6)	55.8 (12.6)	
Sex			.8033			.8917
Female	45 (38.8)	27 (37.0)		44 (38.9)	45 (39.8)	
Male	71 (61.2)	46 (63.0)		69 (61.1)	68 (60.2)	
Race						.5090
White	100 (86.2)	67 (91.8)	.3668	97 (85.8)	98 (86.7)	
Black/African American	2 (1.7)	0 (0.0)		2 (1.8)	0 (0.0)	
Asian	6 (5.2)	1 (1.4)		6 (5.3)	5 (4.4)	
Other	8 (6.9)	5 (6.9)		8 (7.1)	10 (8.9)	
Time from initial diagnosis to 1L start			.0973			.9098
Q1	25 (21.6)	23 (31.5)		24 (21.2)	23 (20.4)	
Q2	25 (21.6)	21 (28.8)		25 (22.1)	26 (23.0)	
Q3	31 (26.7)	17 (23.3)		30 (26.6)	34 (30.1)	
Q4	35 (30.2)	12 (16.4)		34 (30.1)	30 (26.5)	
Stage at initial diagnosis			.5781			.7337
Stage I-IIIA	4 (3.5)	1 (1.4)		4 (3.5)	5 (4.4)	
Stage IIIB-IV	109 (94.0)	71 (97.3)		109 (96.5)	108 (95.6)	
Unknown	3 (2.6)	1 (1.4)		0 (0.0)	0 (0.0)	
ECOG Performance Status			.8147			.7010
0/1	112 (96.6)	70 (95.9)		109 (96.5)	110 (97.4)	
2	4 (3.4)	3 (4.1)		4 (3.5)	3 (2.6)	
Smoking Status, no	67 (57.8)	45 (61.6)	.5966	65 (57.5)	66 (58.4)	.8928
Brain metastases, yes	3 (2.6)	7 (9.6)	**.0363**	3 (2.7)	1 (0.9)	.3130

Abbreviations: ECOG, European Cooperative Oncology Group; n, number of patients in sub-group; Q1, 1st quartile; Q2, 2nd quartile; Q3, 3rd quartile; Q4, 4th quartile; 1L, first-line therapy; SD, standard deviation.

Notes: Statistical comparisons between treatment arms were computed using t-tests for continuous variables and Chi-square and Fisher's exact test for categorical variables.

Table S6. Baseline characteristics among patients with *RET*-mutated MTC – Subgroup 2

(n [%]) unless specified	Pre-matched			Genetic matching		
	Selpercatinib arm (n = 116)	Comparator arm (n = 136)	p-value	Selpercatinib arm (n = 113)	Comparator arm (n = 113)	p-value
Age at index date, year						
Mean (SD)	55.8 (15.5)	53.8 (15.8)	.3194	55.7 (15.6)	55.3 (13.3)	.8451
Sex						
Female	45 (38.8)	48 (35.3)		44 (38.9)	44 (38.9)	
Male	71 (61.2)	88 (64.7)	.5662	69 (61.1)	69 (61.1)	1.0000
Race						
White	100 (86.2)	123 (90.4)		97 (85.8)	97 (85.8)	
Black/African American	2 (1.7)	1 (0.7)		2 (1.8)	2 (1.8)	
Asian	6 (5.2)	2 (1.5)		6 (5.3)	6 (5.3)	
Other	8 (6.9)	10 (7.4)	.3392	8 (7.1)	8 (7.1)	1.0000
Time from initial diagnosis to 1L start						
Q1	23 (19.8)	40 (29.4)		22 (19.5)	21 (18.6)	
Q2	27 (23.3)	36 (26.5)		27 (23.9)	29 (25.7)	
Q3	26 (22.4)	37 (27.2)		25 (22.1)	26 (23.0)	
Q4	40 (34.5)	23 (16.9)	**.0125**	39 (34.5)	37 (32.7)	.9827
Stage at initial diagnosis						
Stage I-IIIA	4 (3.5)	7 (5.2)		4 (3.5)	3 (2.7)	
Stage IIIB-IV	109 (94.0)	126 (92.7)		109 (96.5)	110 (97.3)	
Unknown	3 (2.6)	3 (2.2)	.7931	0 (0.0)	0 (0.0)	.7010
ECOG Performance Status						
0/1	112 (96.6)	126 (92.7)		109 (96.5)	109 (96.5)	
2	4 (3.5)	10 (7.4)	.1774	4 (3.5)	4 (3.5)	1.0000
Smoking Status, no	67 (57.8)	86 (63.2)	.3086	65 (57.5)	66 (58.4)	.8928
Brain metastases, yes	3 (2.6)	10 (7.4)	.0882	3 (2.7)	3 (2.7)	1.0000

Abbreviations: ECOG, European Cooperative Oncology Group; n, number of patients in sub-group; Q1, 1st quartile; Q2, 2nd quartile; Q3, 3rd quartile; Q4, 4th quartile; 1L, first-line therapy; SD, standard deviation.

Notes: Statistical comparisons between treatment arms were computed using t-tests for continuous variables and Chi-square and Fisher's exact test for categorical variables.

Table S7. Genetic matching investigator-assessed outcomes among patients with *RET*-mutated MTC – Subgroups 1 and 2

	Subgroup 1			Subgroup 2		
	**Selpercatinib arm (n = 113)**	**Comparator arm (n = 113)**	**p-value**	**Selpercatinib arm (n = 113)**	**Comparator arm (n = 113)**	**p-value**
**TTD**						
Median (95% CI), month	NR (55.3, NR)	16.5 (12.0, 24.9)		NR (55.3, NR)	17.3 (12.6, 24.1)	
HR (95% CI)	0.18 (0.12, 0.27)		**<.0001**	0.17 (0.12, 0.26)		**<.0001**
**TTNT-D**						
Median (95% CI), month	NR (NR, NR)	19.0 (16.0, 29.3)		NR (NR, NR)	21.5 (14.0, 24.7)	
HR (95% CI)	0.13 (0.08, 0.21)		**<.0001**	0.12 (0.08, 0.20)		**<.0001**
**TTP**						
Median (95% CI), month	NR (NR, NR)	18.5 (13.8, 29.3)		NR (NR, NR)	18.0 (13.0, 23.8)	
HR (95% CI)	0.18 (0.12, 0.28)		**<.0001**	0.17 (0.11, 0.26)		**<.0001**
**ORR**						
N (%)	95 (84.1)	22 (19.5)		95 (84.1)	16 (14.2)	
OR (95% CI)	21.83 (11.00, 43.35)		**<.0001**	32.00 (15.41, 66.42)		**<.0001**

Abbreviations: CI, confidence interval; HR, hazard ratio; n, number of patients in sub-group; TTD, time to treatment discontinuation; TTNT-D, time to next treatment or death; TTP, time to progression.

Table S8. Baseline characteristics among patients with *RET* fusion-positive TC – Subgroup 1

(n [%]) unless specified	Unadjusted		
	**Selpercatinib arm (n = 24)**	**Comparator arm (n = 15)**	**p-value**
Age at index date, year			
Mean (SD)	57.7 (16.5)	59.0 (17.7)	.8126
Sex			
Female	10 (41.7)	10 (66.7)	
Male	14 (58.3)	5 (33.3)	.1286
Race			
White	18 (75.0)	5 (33.3)	
Black/African American	0 (0.0)	2 (13.3)	
Asian	1 (4.2)	8 (53.3)	
Other	5 (20.8)	0 (0.0)	**.0009**
Time from initial diagnosis to 1L start			
Q1	3 (12.5)	6 (40.0)	
Q2	8 (33.3)	2 (13.3)	
Q3	8 (33.3)	2 (13.3)	
Q4	5 (20.8)	5 (33.3)	.0910
Stage at initial diagnosis			
Stage I-IIIA	0 (0.0)	0 (0.0)	
Stage IIIB-IV	24 (100.0)	14 (93.3)	
Unknown	0 (0.0)	1 (6.7)	.2000
ECOG performance status			
0/1	23 (95.8)	15 (100.0)	
2	1 (4.2)	0 (0.0)	.4232
Smoking Status, no	12 (50.0)	10 (66.7)	.4889
Brain metastases, yes	1 (4.2)	0 (0.0)	.0140

Abbreviations: ECOG, European Cooperative Oncology Group; n, number of patients in sub-group; Q1, 1st quartile; Q2, 2nd quartile; Q3, 3rd quartile; Q4, 4th quartile; 1L, first-line therapy; SD, standard deviation.

Notes: Statistical comparisons between treatment arms were computed using t-tests for continuous variables and Chi-square and Fisher's exact test for categorical variables.

Table S9. Unadjusted investigator-assessed outcomes among patients with *RET* fusion-positive TC – Subgroup 1

	**Subgroup 1**		
	Selpercatinib arm (n = 24)	Comparator arm (n = 15)	p-value
**TTD**			
Median (95% CI), month	NR (NR, NR)	11.1 (6.3, 36.1)	
HR (95% CI)	0.15 (0.05, 0.41)		**<.0001**
**TTNT-D**			
Median (95% CI), month	NR (NR, NR)	12.0 (8.8, NR)	
HR (95% CI)	0.18 (0.06, 0.54)		**<.0001**
**TTP**			
Median (95% CI), month	NR (38.5, NR)	13.6 (7.1, NR)	
HR (95% CI)	0.18 (0.06, 0.54)		**<.0001**
**ORR**			
N (%)	20 (83.3)	8 (53.3)	
OR (95% CI)	4.38 (1.00, 19.2)		**.0428**

Abbreviations: CI, confidence interval; HR, hazard ratio; n, number of patients in sub-group; TTD, time to treatment discontinuation; TTNT-D, time to next treatment or death; TTP, time to progression.
